# Bayesian Methods for Medical Test Accuracy

**DOI:** 10.3390/diagnostics1010001

**Published:** 2011-05-05

**Authors:** Lyle D. Broemeling

**Affiliations:** Broemeling & Associates Inc., 1023 Fox Ridge Road, Medical Lake, WA 99022, USA; E-Mail: broemeli2@aol.com

**Keywords:** Bayesian inference, posterior distribution, prior distribution, ROC curve, verification bias, tests without a gold standard

## Abstract

Bayesian methods for medical test accuracy are presented, beginning with the basic measures for tests with binary scores: true positive fraction, false positive fraction, positive predictive values, and negative predictive value. The Bayesian approach is taken because of its efficient use of prior information, and the analysis is executed with a Bayesian software package WinBUGS®. The ROC (receiver operating characteristic) curve gives the intrinsic accuracy of medical tests that have ordinal or continuous scores, and the Bayesian approach is illustrated with many examples from cancer and other diseases. Medical tests include X-ray, mammography, ultrasound, computed tomography, magnetic resonance imaging, nuclear medicine and tests based on biomarkers, such as blood glucose values for diabetes. The presentation continues with more specialized methods suitable for measuring the accuracies of clinical studies that have verification bias, and medical tests without a gold standard. Lastly, the review is concluded with Bayesian methods for measuring the accuracy of the combination of two or more tests.

## 1. Introduction

This review presents and describes the Bayesian techniques that are available for estimating the accuracy of various medical tests used in the diagnosis and treatment of disease, with a primary focus on cancer. Fundamental measures of test accuracy are first introduced, and include the true positive rate (sensitivity), the false positive rate (1-specificity), the positive and negative predictive values, and the area under the ROC curve. Bayesian approaches are very efficient because they are based on prior information that is readily available from previous related studies.

Estimating the test accuracy when verification bias is present is considered next. Verification bias occurs when not all of the patients are subject to a gold standard. For example, consider mammography, where those patients that test positive are usually referred to the gold standard (pathology), but where those that test negative are usually not referred to pathology. Or consider PSA (prostate specific antigen) testing for prostate cancer, where those that test negative are not usually subject to the gold standard (pathology). When verification bias is present, there are special biostatistical methods that are available for providing unbiased estimates of test accuracy.

There are many cases, where a gold standard is not available for estimating test accuracy, but there is an imperfect reference standard. For example there may be two tests, one a so-called new test and the other the imperfect reference standard used to diagnose a bacterial infection, but no gold standard is available. Relative to the imperfect reference standard, the accuracy of the new test can be estimated, however, such estimates can be misleading, but fortunately, there are statistical procedures that are available for ‘correcting’ the estimated accuracy of the new test.

This review involves many of the tests used in medical practice for the diagnosis and monitoring of disease. Of the many tests used in medicine, many are based on imaging devices such as X-ray, CT(computed tomography), MRI(magnetic resonance imaging), mammography, nuclear medicine (PET(positron emission tomography) and SPECT(single-photon emission computed tomography) gamma cameras), and ultrasound. Of course, there are many others based on biomarkers, such as PSA (prostate specific antigen) for prostate cancer, CA19-9 and CA125 for pancreatic disease, and blood glucose values for diabetes.

What biostatistical methods will be described in this review? When estimating the accuracy with the fundamental measures such as the true and false positive rates and the positive and negative predictive values, ratios or fractions (sometimes referred to as rates) will be employed. On the other hand, when estimating the area under the ROC curve, more advanced methods will be explained and illustrated with various examples. Special methods have been developed for estimating the accuracy of medical tests when verification bias is present and these will be illustrated with several examples, as will the case when there is no gold standard but an imperfect reference standard is available.

When the study and patient covariates are taken into account, regression methods provide the way to estimate medical test accuracy. For example, in screening for breast cancer, the patient's age and use of hormones have an effect on breast cancer incidence and should be taken into account when estimating the accuracy of mammography.

Another aspect of test accuracy to be described is the role agreement plays in estimating the accuracy of a medical test. There are usually several observers or readers involved in observing the medical test results and each has their own interpretation of the test outcomes. For example, suppose three radiologists are interpreting the same CT image in order to diagnose lung cancer metastasis, then there could be disagreement as to the degree of metastasis of the disease. There are many studies where there are separate estimates of test accuracy corresponding to the several readers of the test results, and this review will present methods for estimating the agreement between the readers.

## 2. Sources of Information

The author will base the review on two sources of information, textbooks and articles in the statistical and medical literature. There are three textbooks that are devoted to statistical methods for the estimating test accuracy and they are: Broemeling [1], who develops methods based on a Bayesian approach, and Pepe [2] and Zhou, Obuchowski, and McClish [3] who for the most part, use non Bayesian methods such as maximum likelihood *etc.* Many of the methods and examples for this review are taken from these books, while others are based on articles in the medical literature. For example, some examples are based on articles in *Radiology* and others on the *Journal of Pathology*.

## 3. Basic Measures of Test Accuracy

The basic measures of test accuracy are computed from the information in the 2 by 2 table below.

The *n_ij_* are the number of subjects with test score i = 0 or 1 and disease status j = 0 or 1, while *θ_ij_* is the corresponding probability, where D = 0 indicates no disease and D = 1 indicates disease. *θ_ij_* is the probability a patient has test score i and disease status j, where i,j = 0 or 1, thus *n*_00_ is the number of patients without disease and a negative test score X = 0. The true and false positive fractions TPF and FPF are defined as

(1) $$\text{TPF}(\theta )=\theta_{11}/(\theta_{11}+\theta_{01})=\text{P}(\text{X}=1|\text{D}=1)$$

and

(2) $$\text{FPF}(\theta )=\theta_{10}/(\theta_{00}+\theta_{10})=\text{P}(\text{X}=1|\text{D}=0)$$

Thus, the true positive fraction is the proportion of patients with disease who test positive and the false positive fraction is the proportion of non-diseased individuals who test positive for disease. Note that these two measures of accuracy are defined in terms of the unknown cell probabilities of the above 2 by 2 table. Note each cell probability *θ_ij_* is estimated by the corresponding fraction *n_ij_ / n*, where n is the total number of individuals in the study. Usually the study is designed as follows: the individuals are selected at random from a well-defined population, such that the cell frequencies follow a multinomial distribution, and consequently the variance or standard deviation of the estimator *n_ij_ / n* of *θ_ij_* is known. If one takes a Bayesian approach assuming a uniform prior distribution for the cell probabilities, it is known that the posterior distribution of the cell probabilities is Dirichlet with parameter vector

(3) $$\theta /\text{data}\sim \text{Dir}(n_{00}+1,n_{01}+1,n_{10}+1,n_{11}+1)$$

By data is meant the totality of the cell frequencies of the above table. As an example, consider the pexample examined by Pepe [2] based on the study by Weiner *et al.* [4], which is a cohort study of 1465 subjects, where each is classified as to disease status (coronary artery disease (CAD) via an angiogram) and a diagnostic test, the exercise stress test (EST), which is a nuclear medicine procedure and data can be found in Pepe [2].

What are the sensitivity and specificity of the exercise stress test? The Bayesian uses the posterior distribution (3) of the cell probabilities to estimate the true and false positive fractions (1) and (2) by generating samples from the posterior distribution of (1) and (2). Note (1) and (2) are functions of the cell probabilities, thus the posterior distribution of the true positive fraction is determined by generating samples from the Dirichlet distribution (3), then transforming those samples to samples from the true positive fraction via the formula (1). I used 55,000 samples generated from the Dirichlet distribution (3) then transformed each one via formula (1) to get the 55,000 observations from the true positive fraction.

The software package I used is WinBUGS® which is an object-oriented language specifically designed for making Bayesian inferences where the samples from the posterior distribution are generated via Monte Carlo Markov Chain (MCMC) techniques, and the reader is referred to Woodworth [5] for additional information about such simulation methods.

Now returning to the exercise stress test of Table 1, what are the Bayesian estimates of the true and false positive fractions?

Table 3 reports the WinBUGS output for making inferences about the true and false positive fractions, and it is seen that the mean of the posterior distribution of the TPF is 0.796 and that the standard deviation of the posterior distribution of TPF is 0.0125. A 95% credible interval for the TPF is (0.7716, 0.8208) and the median of the posterior distribution is 0.7967 implying that the posterior distribution of the TPF is symmetric about the mean 0.7967. The error column of the above table gives one information about the accuracy of using 55,000 observations to estimate the ‘true’ posterior mean. An important aspect of the package is that it generates plots of the various posterior distribution, as for example for the FPF given by:

Note that the density is centered over the posterior mean of 0.2612 and appears to be symmetric about the mean. My impression is that the exercise stress test is accurate if one uses the sensitivity to estimate accuracy, but I am not so sure about the relatively high value for the false positive fraction.

There are other ways to measure the accuracy of the exercise stress test, namely the positive and negative predictive values:

(4) $$\text{PPV}(\theta )=\theta_{11}/(\theta_{01}+\theta_{11})=\text{P}(\text{D}=1|\text{X}=1)$$

and

(5) $$\text{NPV}(\theta )=\theta_{00}/(\theta_{00}+\theta_{01})=\text{P}(\text{D}=0|\text{X}=0)$$

These measures of accuracy are of interest to the patient. Take for example the positive predictive value (PPV), which is the proportion of patients who test positive that have the disease as determined by the gold standard), and the negative predictive value of NPV, which is defined as the proportion of patients that test negative that do not have the disease.

A casual look at the above table tells me that the exercise stress test is accurate based on the PPV, but not on the NPV. Among those that test negative, approximately 61% do not have the disease! Does this give you confidence in the exercise stress test? It should be noted that rarely is a test perfect, where the TPF, FPF, PPV, and NPV are all one!

Consider the diagnostic likelihood ratios as a third group of test accuracy measures and are defined as the positive diagnostic likelihood ratio

(6) $$\begin{array}{c} \text{PDLR}(\theta )=\text{P}(\text{X}=1|\text{D}=1)/\text{P}(\text{X}=1|\text{D}=0)\\ =[\theta_{11}/(\theta_{11}+\theta_{01})]/[\theta_{10}/(\theta_{10}+\theta_{00})]\\ =\text{TPF}(\theta )/\text{FPF}(\theta ) \end{array}$$

and the negative diagnostic likelihood ratio

(7) $$\begin{array}{cc} \text{NDLR}(\theta ) & =\text{P}(\text{X}=0|\text{D}=1)/\text{P}(\text{X}=0|\text{D}=0)\\ & =[\theta_{01}/(\theta_{11}+\theta_{01})]/[\theta_{00}/(\theta_{10}+\theta_{00})]\\ & =\text{FNF}(\theta )/\text{TNF}(\theta ) \end{array}$$

These measures are quite different than that the previous measures. The positive diagnostic likelihood ratio is a fraction, the numerator of which is the TPF and the denominator is the FPF, and note that larger values indicate a more accurate test, because more accurate tests have larger TPF and smaller FPF. As for the negative diagnostic likelihood ratio, smaller values are indicative of a more accurate test, because more accurate tests have a smaller FNF and a larger TNF! With regard to the exercise stress test, the Bayesian analysis gives the following results:

Is the PDLR large enough? Recall that the TPF is three times that of the FPF and the FNF is 0.27 that of the TNF. For me the TPF, FPF, FNF, and TNF each gives a separate way to view the accuracy of a medical test and are more informative than the diagnostic likelihood ratios.

The ROC curve is another way to measure the accuracy of a medical test and is appropriate when the test scores are ordinal or continuous. Consider the results of mammography given to 60 women, of which 30 had the disease. This is presented in Zhou *et al.* ([3], p. 21).

The radiologist assigns a score from 1–5 to each mammogram, where 1 indicates a normal lesion, 2 a benign, 3 a lesion which is probably benign, 4 indicates suspicious, and 5 malignant. How would one estimate the accuracy for mammography from this information? When the test results are binary, the observed TPF and FPF are calculated, but here there are 5 possible results for each image. The scores could be converted to binary by designating 4 as the threshold, then scores 1–3 are negative and 4–5 are positive test results. Then estimate the TPF as tpf = 23/30 and the specificity (1-FPF) as (1-fpf) = 21/30. Another approach would be to use each test result as a threshold and calculate the tpf and fpf, which are depicted in Table 6.

**Figure 2:**
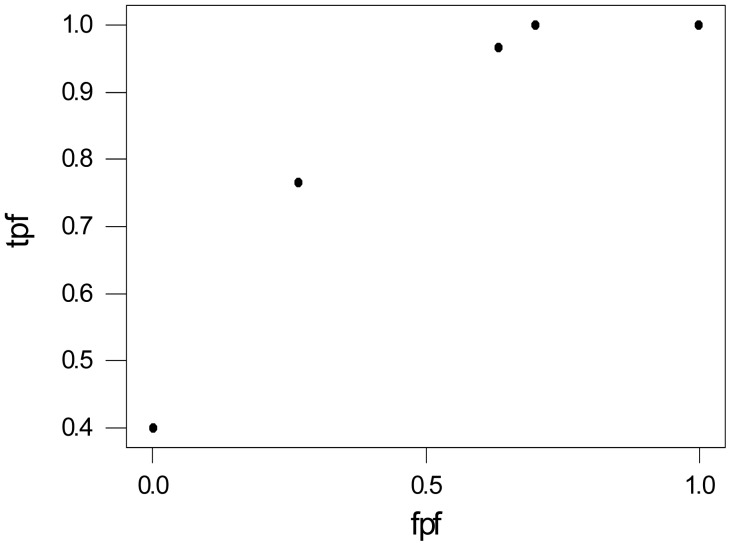
Empirical ROC for Mammography.

Of the 30 diseased, 30 had a score of at least 1, while 23 had a score of at least 4. On the other hand, of the 30 without cancer, 30 had a score of at least 1, and 8 had a score of at least 4, *etc.* Figure 2 is a plot of the observed true and false positive values of Table 6. What does this graph tell us about the accuracy of mammography?

The area under the ROC gives the intrinsic accuracy of a diagnostic test and can be interpreted in several ways. Either as the average sensitivity for all values of specificity, or the average specificity for all values of sensitivity, or as the probability that the diagnostic score of a diseased patient is more of an indication of disease than the score of a patient without the disease or condition. The problem is in determining the area under the curve. For the graph above, there are five points corresponding to the five threshold values.

In the case of ordinal data, the area under the curve (AUC) as determined by a linear interpolation of the points on the graph (including (0,0) and (1,1)) and the area has the following interpretation,

(8) AUC=P(Y>X)+(1/2)P(Y=X)

See the description of Pepe ([2], p. 92), where it is assumed that one patient is selected at random from the population of diseased patients, with a diagnostic score of Y and another patient, with a score of X, is selected from the population of non-diseased patients. Note that the AUC depends on the parameters of the model. Let us return to the mammography example and estimate the area under the curve via a Bayesian method.

For the mammography example, the area is defined as

(9) AUC(θ,ϕ)=P(Y>X∣θ,ϕ))+(1/2)P(Y=X∣θ,ϕ)

where Y (= 1,2,3,4,5) is the diagnostic score for a person with breast cancer and X (= 1,2,3,4,5) for a person without. It can be shown

(10) $$\text{AUC}(\theta ,\phi )=\sum_{i=2}^{i=5}\sum_{j=1}^{j=i-1}\theta_{i}\phi_{j}+(1/2)\sum_{i=1}^{i=5}\theta_{i}\phi_{i}$$

It is assumed the Y and X are independent, given the parameters, and that P(Y = i) = *θ_i_* and P(X = j) = *ϕ_j_*, i, j = 1,2,3,4,5. AUC is a parameter that depends on the parameters *θ* and *ϕ*, and their posterior distributions are *θ / data* ∼ *Dir*(2,1,7,12,13) and independent of *ϕ / data* ∼ *Dir*(10,3,12,9,1), assuming a uniform prior for the parameters, see Table 5.

Samples from the posterior distribution of the AUC are generated by sampling from the posterior distributions of *θ* and *ϕ*. This is accomplished with WinBUGS, where 55,000 observations are generated from the posterior distribution of all the parameters.

Notice that mammography gives fair to good accuracy based on the ROC area, which is estimated as 0.7811(0.0514) with the posterior mean and by (0.6702,0.8709) using a 95% credible interval. The Bayesian estimate of the ROC area is similar to the Zhou *et al.* ([3], P. 30) estimate. The MCMC error for the parameter based on 50,000 observations is less than 0.001, but the reader should vary the simulation sample size to see its effect on the MCMC error and posterior mean. The parameter A1 is P[Y > X] and estimated as 0.688(0.06350) and the probability of a tie, P[Y = X], given by A2, is estimated as 0.1861(0.0307).

See Broemeling ([1], p. 82) and Zhou ([3], p. 134) with an example from mammography. In mammography the mammogram is partitioned into five areas of interest and the radiologist assigns a score from say 1 to 5 (which indicates the degree of malignancy) as in the above example of mammography in Table 5, and one would expect the scores to be correlated between the five areas of interest, which is taken into account by the Bayesian approach.

With regard to continuous test scores, Bayesian estimators of the ROC area are easily determined by the WinBUGS code of O'Malley *et al.* [6]. The area under the ROC curve gives an intrinsic value to the accuracy of a diagnostic test and has a long history beginning in signal detection theory. See Egan [7] for the early use of the ROC curve in signal detection theory. Also, the books by Pepe [2] and Zhou *et al.* [3] provide the history as well as the latest statistical methods (non Bayesian) for using ROC curves in diagnostic medicine. The ROC area is generally accepted as the way to measure diagnostic accuracy in radiology.

Let X be a quantitative variable and r a threshold value, and consider the test positive when X ≥ r, otherwise negative, then the ROC curve is the set of all points

(11) $$\begin{array}{cc} \text{ROC}(.) & =\{[\text{FPF}(\text{r}),\text{TPF}(\text{r})],\text{r any real number}\}\\ & =\{[\text{t},\text{ROC}(\text{t})],\text{t}\in (0,1)\} \end{array}$$

where t = FPF(r), that is, r is the threshold corresponding to t. As r becomes large, FPF(r) and TPF(r) tend to zero, while if r becomes small, FPF(r) and TPF(r) tend to 1, thus the ROC curve passes through (0,0) and (1,1). If the area under the curve is 1, the test is discriminating perfectly between the diseased and non-diseased groups, while if the area is 0.5, the test cannot discriminate between the two groups.

Pepe ([2], ch. 4) presents several useful properties of the ROC curve, namely: (1) the invariance of the ROC curve under monotone increasing transformations of X, (2) interpreting the ROC area for continuous variables as AUC = P(X > Y), and (3) a formula for the AUC area when X is normally distributed. The Bayesian approach to estimating the ROC area is based on

(12) $$\text{AUC}=\Phi [a/\sqrt{1+b^{2}}]$$

where X is normally distributed,

(13) $$\text{a}=(\mu_{D}-\mu_{\bar{D}})/\sigma_{D}$$

and

(14) $$\text{b}=\sigma_{D}/\sigma_{\bar{D}}$$

The mean and standard deviation of X for the diseased population are *μ_D_* and *σ_D_* respectively, while *μ_D̄_* and *σ_D̄_* are the mean and standard deviation of X for the non-diseased. Φ is the cumulative distribution function of the standard normal distribution. Formula (12) is the binormal assumption and is cited by many authors, including Pepe [2], who presents a good discussion of its use. Note that the ROC area AUC depends on the unknown parameters of the model.

Bayesian methods for estimating the ROC area with continuous data will be illustrated by referring to a hypothetical example of diabetes, which involves 59 subjects with diabetes and 19 without, where those with diabetes have a mean blood glucose value of 123.34 mg/dl and those without have a mean value of 107.54. The corresponding standard deviations are 6.76 for those with diabetes and 9.09 mg/dl for those without the disease, and the actual values from the study are given below in the first list statement of the WinBUGS program appearing below. The y vector of the first list statement contains the blood glucose values, where the first 49 entries correspond to diabetic patients and the remaining 19 to non diabetic patients. Note the first 49 entries of the d vector are 1 designating a diabetic patient, while the 19 remaining entries are zero. The O'Malley *et al.* [6] approach assumes binormality, where the blood glucose values for both the diabetic and non diabetic patients are assumed to be normally distributed. I have inserted comments about the WinBUGS code designated by a # symbol.

### WinBUGS Code for Diabetes Example


model;# Calculates posterior distribution of model parameters and the area under curve. y = test# Based on O'Mally *et al.* [6] regression method.{# likelihood function  for(i in 1:N) {# The following statement is the regression of y on the disease vector d   y[i]∼dnorm(mu[i],precy[d[i]+1]);#   yt[i] < −log(y[i]); # logarithmic transformation# The beta vector are the regression coefficients   mu[i] < −beta[1] + beta[2] × d[i];    }# prior distributions - non-informative prior; similarly for informative priors  for(i in 1:P) {   beta[i] ∼ dnorm(0, 0.000001);    }  for(i in 1:K) {   precy[i]∼dgamma(0.001, 0.001);   vary[i] < −1.0/precy[i];    }# calculates area under the curve  la1 < −beta[2]/sqrt(vary[1]); # ROC curve parameters  la2 < −vary[2]/vary[1];# auc is the area under the ROC curve  auc < −phi(la1/sqrt(1+la2));}# Diabetes datalist(K = 2, P = 2, N = 78, y = c(123,129,115,131,119,111,129,127,118,111, 131,118,126,130,122,112,122,128,123,119,132,118,126,136,118,122, 119,117,129,120,125,115,131,123,130,113,128,138,119,118,124,127, 139,120,122,120,114,114,122,127,123,118,131,130,139,125,135,121,124, 109,106,100,88,106,108,110,111,112,94,122,110,113,106,114,101,99,128,106), d = c(1,1,1,1,1,1,1,1,1,1,1,1,1,1,1,1,1,1,1,1,1,1,1,1,1,1,1,1,1,1,1,1,1,1,1,1,1,1, 1,1,0,0,0,0,0,0,0,0,0,0,0,0,0,0,0,0,0,0,0))# the initial values for the simulationlist(beta = c(0,0),precy = c(1,1))

There are 78 patients, of which 19 do not have diabetes. The primary parameters are the area under the curve, auc, and the regression coefficients. Based on the above code, 75,000 observations are generated from the posterior distribution for the area and regression parameters.

**Figure 3:**
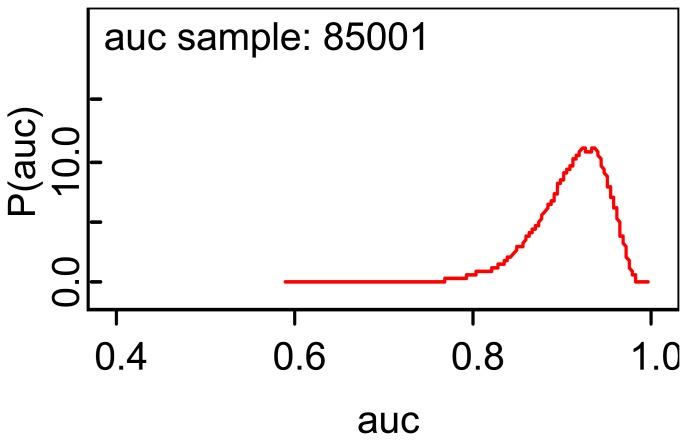
Posterior density of ROC area for head trauma study.

The estimated area is 0.9082 which implies that the blood glucose test had good accuracy and the area given is almost identical to that given by the basic formula (12). A plot of the posterior density is shown in Figure 3. The second regression coefficient is estimated as 16.14, which implies that the group effect is strong on the mean blood glucose values, which is one reason why the ROC area is as high as it is. Also, note the variation in the MCMC errors of estimation.

The plot indicates a slight asymmetry which is also implied by comparing the posterior median with the posterior mean.

This review is continued by considering two medical tests that are each applied to all patients, and a good example of this is the following hypothetical example of CT and MRI imaging of subjects for lung cancer. In order to compare the two, consider the following two tables, the first for lung cancer patients and the other for those without lung cancer. The example is employed to illustrate the Bayesian estimation of the basic measures of test accuracy and to compare the two modalities in regard to the true positive and false positive fractions. There are 995 subjects with the disease and 435 without lung cancer and the important question is which modality, MRI or CT is most accurate and by how much? Note that both modalities are imaging the same subjects, and one would expect the MRI and CT test scores to be correlated!

The Bayesian analysis will consist of finding the posterior distribution of the true and false positive fractions of the two modalities and comparing them on the basis of the ratios of the two basic measures. Let *θ_ij_* be the probability that a lung cancer patient has a CT score of i and an MRI score of j, where i, j = 0,1, where 0 indicates a negative outcome and 1 a positive. In a similar manner, let *ϕ_ij_* be the corresponding probability for a non-diseased subject.

Assuming a uniform prior distribution for *θ* = (*θ*_00_, *θ*_01_, *θ*_10_, *θ*_11_) and *ϕ* = (*ϕ*_00_, *ϕ*_01_, *ϕ*_10_, *ϕ*_11_), their joint posterior distribution is Dirichlet with parameter (23,192,31,753;149,168,50,72). The analysis is executed with 55,000 observations generated from the joint posterior distribution of the cell probabilities *θ_ij_* and gives the following results:

Note that fpfct is the false positive fraction for CT while rfpf (ct/mri) is the ratio of the false positive fraction of CT to that of MRI, and the 95% credible intervals for the two ratios do not include 1, implying that the two modalities have different accuracies for diagnosing lung cancer. With regard to the true positive fraction, MRI is more accurate, but CT has the smallest false positive ratio, and in fact the false positive fraction for MRI is quite large with a posterior median of 0.5469. Which modality would you use? I would use both.

The above approach can be extended to comparing the ROC areas of two modalities and more information can be found in Broemeling ([1], p. 84).

## 4. Verification Bias

Up to this point, interest has been confined to standard studies of medical test accuracy, but now attention will be focused on specialized methods for measuring accuracy. In a standard study, each subject will have been subjected to the gold standard where the disease status is known, but there are many studies where this is not possible. For example with the exercise stress test, those that test positive will most likely be referred to the gold standard (coronary angiography), however those that test negative will not, unless there are other indicators that point to disease. Actually, verification bias is present in many medical test accuracy studies; however, often the investigator is unaware that bias is present. According to Zhou *et al.* [3], Greenes and Begg [8] reviewed 145 investigations that took place over the period 1976–1980 and found that 26% had verification bias that was not recognized by the authors. In addition, Bates, Margolis, and Evans [9] reported that at least 1/3 of 54 pediatric studies had unrecognized verification bias. There are many more such studies, including those reported by Philbrick, Horwitz, and Feinstein [10] who found that of 33 diagnostic studies for coronary artery disease, 31 had verification bias. In a major review of verification bias, that reviewed 112 studies in major medical journals, Reid, Lachs, and Feinstein [11] reported finding that 54% had verification bias!

This section will present Bayesian methods for estimating test accuracy, when some of those that test positive or negative are referred to the gold standard, which is presented in the following table.

Consider the following table for one binary test Y = 0,1 where verification bias is present.

V = 1 indicates the patient is verified and the disease status is known, and V = 0 indicates a patient has not been verified, thus, there are *u*_1_ individuals who are not verified when Y = 1. The total number of patients in the study are *m*_1_+ *m*_0_, while the number who tested positive and had the disease is *s*_1_. If the test accuracy is based on only the verified patients, the estimates are misleading. Fortunately there are statistical methods for correcting these misleading estimates of test accuracy. In order to implement these procedure, the missing at random assumption (MAR) is imposed, which entails assuming that the decision to verify the disease status depends on only the results Y of the diagnostic test and not other factors related to the disease status. That is to say:

(15) P[V=1∣D,Y]=P[V=1∣Y]

Our approach is likelihood based, where the likelihood function is based on the conditional distribution of the disease status D = 1, given Y = 0 or 1, and on the marginal distribution of Y. The probability that Y = 1, given D = 1 is then found by Bayes theorem. The derivation of the likelihood and relevant posterior distributions is as follows:

Let

(16) $$\phi_{i}=P[D=1|Y=i]$$

and

(17) $$\theta_{i}=P[Y=i]$$

where i = 0,1, then the likelihood function for the parameters is

(18) $$L(\theta ,\phi )\propto \phi_{1}^{s_{1}}{\left(1-\phi_{1}\right)}^{r_{1}}{\phi_{0}}^{s_{0}}{\left(1-\phi_{0}\right)}^{r_{0}}{\theta_{1}}^{m_{1}}{\theta_{0}}^{m_{0}}$$

where all parameters are between zero and one and *θ*_0_ + *θ*_1_ = 1. With a uniform prior for all parameters, the posterior distribution of the parameters is as follows:

(19) $$\phi_{i}\sim \text{beta}(s_{i}+1,r_{i}+1)$$

for i = 0,1, and (*θ*_0_, *θ*_1_) has a Dirichlet with parameters (*m*_0_ + 1,*m*_1_ +1).

The approach to correcting for bias is to use Bayes theorem to compute

(20) P[Y=1∣D=1]=P[D=1∣Y=1]/P[D=1]

where

(21) $$\text{P}[\text{D}=1]=\phi_{1}\theta_{1}+\phi_{0}\theta_{0}$$

(22) $$\text{Let}\;\alpha_{i}=\phi_{i}\theta_{i}/(\phi_{1}\theta_{1}+\phi_{0}\theta_{0})$$

then *α*_1_ is the sensitivity of the test. On the other hand, let

(23) $$\beta_{1}=(1-\phi_{1})\theta_{1}/(1-\phi_{1}\theta_{1}-\phi_{0}\theta_{0})$$

then *β*_1_ is the false positive fraction, that is, the probability that Y = 1, given D = 0.

Once the posterior distribution of the parameters is determined, the posterior distribution of the true and false positive fractions is also determined.

A good example of verification bias is the study of Drum and Christacopoulos [12], which is a hepatic scintigraphy test for liver disease.

This test had two results Y = 0 or 1, where a 1 indicates a positive result for disease. Note that the total number of subjects is 670, with 474 who tested positive, and among those, 150 were not verified for the disease.

Among those who tested negative, 79 were examined by the gold standard, with 31 of those having the disease. The estimated sensitivity based on the verified patients is 298/329 = 0.905, and the estimated false positive rate is 26/74 = 0.35. What are the corrected estimates and how do they differ from these? The Bayesian analysis assumes a uniform prior for the parameters of the likelihood function and is executed with 50,000 observations for the MCMC simulation.

Note the reasonably good accuracy of the scintigraphy test for liver disease, with a sensitivity of 0.83 and a false positive fraction of 0.2139. Recall the estimated sensitivity using the verified cases only (the naïve estimates) is 0.9224 and the false positive fraction is 0.372, thus, the corrected true and false positive rates are smaller than those calculated from the verified cases only. In general if those that test positive are more likely to be verified than those patients that test negative, the naïve estimates (those based on verified cases only) are such that the true and false positive fractions are larger than the unbiased estimators respectively. See Pepe ([2], p. 169) for further details.

The above approach that produces unbiased estimators for the TPF and FPF, is easily extended to two paired binary tests, but will not be presented here; instead the approach is generalized to estimating the area under the ROC curve for medical tests with ordinal test scores. Consider the typical layout for such a test Y with possible values 1, 2,…, k reported below with familiar notation as:

If a uniform prior distribution is deemed appropriate, the posterior distribution of the *ϕ_i_* is beta with parameters *s_i_*+1 and *r_i_*+1 and that for the *θ_i_* is Dirichlet with parameter (*m*_1_ + 1, *m*_2_ +1, …, *m_k_* +1).

In order to compute the area under the ROC, one must compute P[Y = i∣D = 1] and P[Y = i∣D = 0] for all i = 1, 2, …, k, where the first component is represented by Bayes theorem as

(24) $$\begin{array}{ll} \text{P}[\text{Y}=\text{i}|\text{D}=1] & =\text{P}[\text{D}=1|\text{Y}=\text{i}]\text{P}[\text{Y}=\text{i}]/\text{P}[\text{D}=1]\\ & =\phi_{i}\theta_{i}/P[D=1] \end{array}$$

where,

(25) $$P[D=1]=\sum_{i=1}^{i=k}\phi_{i}\theta_{i}$$

On the other hand, the second component is computed as

(26) $$\text{P}[\text{Y}=\text{i}|\text{D}=0]=(1-\phi_{i})\theta_{i}/\text{P}[\text{D}=0]$$

where P[D = 0] = 1 − P[D = 1].

We are now in a position to compute the area under the ROC curve.

Let

(27) $$\alpha_{i}=P[T=i|\text{D}=1]$$

and

(28) $$\beta_{i}=P[T=i|\text{D}=0]$$

for i = 1, 2,‥, k, then the area under the ROC is given by

(29) $$\text{A}=A_{1}+A_{2}/2$$

where

(30) $$A_{1}=\alpha_{2}\beta_{1}+\alpha_{3}(\beta_{1}+\beta_{2})+\ldots +\alpha_{k}(\beta_{1}+\beta_{2}+\ldots +\beta_{k-1})$$

and

(31) $$A_{2}=\sum_{i=1}^{i=k}\alpha_{i}\beta_{i}$$

Formula (29) for the ROC area is given in Broemeling ([1], p. 72).

The example for ordinal test scores is taken from a hypothetical mammography study with 1,509 subjects, where each patient is given a score of Y where Y = 1, 2, 3, 4, s5.

Note the number of unverified cases is 249 out of a total of 1509 subjects. A Bayesian analysis is performed using 55,000 observations for the simulation and the posterior analysis appears in the following table, where the median ROC area is 0.7764.

Generalizations for verification studies are possible in many directions including extensions to several observers and to using patient and study covariates. Also, it is possible to drop the MAR assumption and to estimate test accuracy. The case of extreme verification bias for binary tests is not considered, which is the case where only those that test positive are verified, while none are verified among those that test negative. See Pepe ([2], p. 180), and see Broemeling [1], Pepe [2], and Zhou *et al.* [3] for additional interesting information about the analysis of verification studies

## 5. Tests with an Imperfect Reference Standard

Suppose that a gold standard does not exist, but that test accuracy of a new test will be assessed with an imperfect gold standard. Many cases exist where there is no perfect gold standard. For example, depression is usually determined by a series of questions and observing the behavior of the patient, but such assessments are highly subjective, and there is no one test that will provide a perfect diagnosis. For infectious diseases, a perfect diagnosis can be elusive, where a culture is taken; however, the culture may not contain the infective agent or if the agent is present may not grow in the culture. Pepe [2] gives other examples, including tests for diagnosing cancer and hearing loss. Zhou *et al.* [3] also present various studies, including the diagnosis of a bacterial infection with the stool and serology tests. Their analysis is to use maximum likelihood while Bayesian is the approach taken here. Other examples presented by Zhou *et al.* include two tests for tuberculosis, with the Tine and Mantour tests, at two different sites, while a third example for detecting pleural thickening is performed by X-ray with three readers. Another interesting example of multiple tests is described by Pepe [2], where *chlamydia* bacterial infection is diagnosed with a blood culture, PCR, and ELISA.

Previous work has focused on maximum likelihood estimation and Bayesian. Zhou *et al.* [3] emphasize maximum likelihood and Bayesian. The Bayesian method is based on earlier work by Joseph, Gyorkos, and Coupal [13] who employ an augmented data approach. The augmented data approach views the missing data (the disease status D of a patient) as an unobservable random variable that can be modeled in such a way as to provide the posterior density of the measures of disease accuracy (true and false positive rates). Such an approach will be used here, because the Bayesian method has the advantage of using prior information and being able to separate the parameters of interest from nuisance parameters. Fortunately, prior information is available for diagnostic tests, especially the disease rates and the accuracy assessments of medical tests, and can be used as part of the posterior analysis.

With the Bayesian approach of Joseph, Gyorkos, and Coupal [13] and Dendukuri and Joseph [14], the various tests are assumed to be conditionally independent, an assumption that will be used in the present approach, however, the assumption will be relaxed in some cases and the two ways compared in estimating test accuracy.

Pepe ([2], p. 195) presents the following example of using an imperfect reference standard R to assess the accuracy of a new test T, namely:

The new test T has a ‘true’ sensitivity of 0.80 (80/100) and a specificity of 0.70 (70/100) but of course this is actually not known because there is no gold standard. Relative to the reference test R, the sestimated sensitivity is also 0.8 (64/80) but has a specificity of 0.61(74/120), thus, the new test is assessed to be less specific than it actually is. Also, with respect to the gold standard, the prevalence of disease is 50%, but is estimated to be 40% with regard to R. Remember the gold standard is not present, we do not know the ‘true’ measures of accuracy, only those with regard to the reference standard can be estimated, and can be misleading!

The two tests are said to be conditionally independent if

(32) P[T,R∣D]=P[T∣D]P[R∣D]

a condition which is usually employed with both the conventional and Bayesian approaches. Using this assumption, Pepe ([2], p. 195) states that it is likely that both the observed (relative to the reference test R) sensitivity and specificity will be decreased.

Are there methods that will improve on the measures of accuracy provided by the imperfect standard test? Using primarily the Bayesian approach, this question will be explored in this chapter. In what is to follow, the subject is introduced with two binary tests, one is the reference test R and the other a new one T whose accuracy is to be assessed. Note none of the patients will have their true disease status D measured, instead each patient will be given a positive or negative score by both tests. A Bayesian approach is taken, where based on the likelihood function the posterior distribution of the sensitivity and specificity are determined. The likelihood function is presented where the missing disease status is modeled by augmented or latent variables. Conditional independence is assumed.

With the likelihood function based on latent variables and assuming conditional independence, the posterior distribution of the sensitivity, specificity, and disease prevalence are determined. An example er analyzed by Joseph, Gyorkos, and Coupal [13] involves a bacterial infection of immigrants to Canada and employs the augmented data method to estimate the sensitivity and specificity of the reference test R (a serology test) and another test T, the stool examination.

Consider a layout for the experiment with the two tests R and T, using the augmented data approach.

When D = 1, the results of the study are:

where the augmented data is represented by the *y_ij_* and the observations by the corresponding *n_ij_*.

Now let

(33) $$\theta_{ij}=P[R=i,T=j|\text{D}=1]$$

i, j=0 or 1, and

(34) $$\phi_{ij}=P[R=i,T=j|\text{D}=0]$$

Then the likelihood function is

(35) $$L(\theta ,\phi /\text{data})\propto p^{y_{‥}}{\left(1-p\right)}^{n_{‥}-y_{‥}}\prod_{i=0}^{i=1}\prod_{j=0}^{j=1}{\theta_{ij}}^{y_{ij}}\prod_{i=0}^{i=1}\prod_{j=0}^{j=1}{\phi_{ij}}^{n_{ij}-y_{ij}}$$

and assuming a uniform prior, the posterior distribution of the parameters p, the *θ_ij_*, and the *ϕ_ij_* can be determined in terms of all the conditional distributions as follows:

If one assumes the conditional independence assumption the likelihood function is expressed directly in terms of the sensitivity and specificity as

(36) $$\begin{array}{c} L(p,s_{1},s_{2},c_{1},c_{2})\propto {s_{1}}^{y_{11}+y_{01}}{\left(1-s_{1}\right)}^{y_{10}+y_{00}}{s_{2}}^{y_{11}+y_{10}}{\left(1-s_{2}\right)}^{y_{01}+y_{00}}\\ {c_{1}}^{n_{10}+n_{00}-y_{10}-y_{00}}{\left(1-c_{1}\right)}^{n_{11}+n_{01}-y_{11}-y_{01}}\\ {c_{2}}^{n_{01}+n_{00}-y_{01}-y_{00}}{\left(1-c_{2}\right)}^{n_{11}+n_{10}-y_{11}-y_{10}}\\ p^{y_{‥}}{\left(1-p\right)}^{n_{‥}-y_{‥}} \end{array}$$

*y*_‥_ is the sum of the *y_ij_* and *n*_‥_ is the sum of the four cell frequencies. The notation has been changed to denote *s*_1_ and *c*_1_ as the sensitivity and specificity of T respectively, while *s*_2_ and *c*_2_ denote the corresponding quantities for the reference R.

For computational purposes and assuming a uniform prior, it is obvious from the above likelihood function that the conditional distribution of the unknown parameters are:

The marginal distribution of p is beta with parameters

(37) $$\begin{array}{c} \text{ap}=y_{‥}+1\\ \text{and}\\ \text{bp}=n_{‥}-y_{‥}+1 \end{array}$$

The conditional distribution of *s*_1_ given the other parameters is beta with parameters as1 and bs1 where

(38) $$\begin{array}{c} \text{as}1=y_{11}+y_{01}+1\\ \text{and}\\ \text{bs}1=y_{10}+y_{00}+1 \end{array}$$

The conditional distribution of *s*_1_, given the other parameters is beta with hyperparameters

(39) $$\begin{array}{c} \text{as}2=y_{11}+y_{10}+1\\ \text{and}\\ \text{bs}2=y_{01}+y_{00}+1 \end{array}$$

The conditional distribution of *c*_1_ is beta with hyperparameters

(40) $$\begin{array}{c} \text{ac}1=n_{10}+n_{00}-y_{10}-y_{00}+1\\ \text{and}\\ \text{bc}1=n_{11}+n_{01}-y_{11}-y_{01}+1 \end{array}$$

and the conditional distribution of *c*_2_ is beta with parameters

(41) $$\begin{array}{c} \text{ac}2=n_{01}+n_{00}-y_{01}-y_{00}+1\\ \text{and}\\ \text{bc}2=n_{11}+n_{10}-y_{11}-y_{10}+1. \end{array}$$

In addition, the posterior distribution of the latent variables is:

The conditional distribution of *y*_11_ given the other variables is binomial with parameters

(42) $$\begin{array}{c} \text{m}11=ps_{1}s_{2}/[ps_{1}s_{2}+(1-p)(1-c_{1})(1-c_{2})](\text{the probability parameter})\\ \text{and}\\ \text{q}11=n_{11} \end{array}$$

The conditional distribution of *y*_10_, given the other parameters is binomial with parameters:

(43) $$\begin{array}{c} \text{m}10=p(1-s_{1})s_{2}/[p(1-s_{1})s_{2}+(1-p)c_{1}(1-c_{2})]\\ \text{and}\\ \text{q}10=n_{10} \end{array}$$

The conditional distribution of *y*_01_, given the other parameters, is binomial with hyperparameters

(44) $$\begin{array}{c} \text{m}01=ps_{1}(1-s_{2})s_{2}/[ps_{1}(1-s_{2})+(1-p)(1-c_{1})c_{2}]\\ \text{and}\\ \text{q}01=n_{01} \end{array}$$

and lastly

The conditional distribution of *y*_00_ given the other parameters is binomial with hyperparameters

(45) $$\begin{array}{c} \text{m}00=p(1-s_{1})(1-s_{2})/[p(1-s_{1})(1-s_{2})+(1-p)c_{1}c_{2}]\\ \text{and}\\ \text{q}00=n_{00} \end{array}$$

It is important to know that the above posterior distributions for the accuracy of two binary tests assumes a uniform prior for *p, s*_1_, *s*_2_, *c*_1_, and *c*_2_ and the assumption of conditional independence between R and T!

An example assuming a uniform prior and conditional independence between an imperfect reference test R and a new test T is presented as follows. Consider the diagnosis of a bacterial infection by *Strongyloides* exposing 162 Cambodian refugees to Canada. They entered Canada from July 1982 to February 1983 and were tested with a Stool examination, which serves as the ‘new’ test T and a serologic reference test R, and the results as reported by Zhou *et al.* ([3], p. 366) are given below.

This information has been analyzed by a number of people, including Joseph, Gyorkos, and Coupal [13] and Dendukuri and Joseph [14]. The observed sensitivity and specificity of the stool exam relative to the serology exam are 38/125 = 0.304 and 35/37 = 0.945 respectively. The main focus is to correct the actual sensitivity and specificity of the stool exam via the methodology derived in the previous section. Assume conditional independence between T and R, then the posterior distribution of the relevant parameters is given by the conditional distributions of each parameter given the others, which are identified in statements (37)–(45).

The above table reports the analysis which is executed with 125,000 observations generated from the posterior distribution of the parameters. As seen from Table 20, the standard deviation for the two sensitivities is almost as large as the mean indicating uncertainty for these measures of accuracy, and the MCMC errors are relatively large (but reasonable) for all parameters. Also, the distributions for *c*_2_ and *s*_1_ are skewed, and I would use the posterior medians to report the accuracy of the two tests.

One can employ the prior information used by Zhou *et al.* ([3], p. 367) who utilized informative prior information about the parameters, namely:

The prior information was elicited form a panel of experts and the ranges of the parameter values converted to the hyperparameters of the corresponding beta prior distribution, that is, a beta prior was used for each parameter with the above values for the parameters of that variable. Note the uncertainty for p, expressed as a range (0,1) and a uniform prior for the prevalence. For example, the prior mean for *c*_1_ the specificity of the stool exam is 0.95, while that for the sensitivity *s*_2_ of the serology test is believe to be 0.80, compared to a prior mean of 0.74 for the sensitivity of the stool exam.

Of course, the accuracy of serology is supposed to be better than that compared to stool, and this is reflected in the prior values of the above table.

A Bayesian analysis is performed utilizing the prior information in the above table and conditional independence between the two tests. Again 125,000 observations are generated from the joint posterior distribution:

Comparing Tables 20 and 22 reveals less uncertainty in the estimates (posterior means) using informative beta priors for the accuracy parameters, and the MCMC errors are much smaller when the informative prior is used. For the accuracy parameters (sensitivity and specificity), the posterior standard deviations are less across the board. Not the posterior distributions appear to be symmetric. This example shows the effect of prior information on the posterior analysis, where a uniform prior was compared to an informative prior (based on expert opinion). Which analysis would you use?

The Bayesian analysis for correcting for an imperfect reference test is easily generalized to multiple binary tests and to the situation where the conditional independence assumption is not imposed. See Broemeling [1], Pepe [2], and Zhou *et al.* [3] for additional information about this interesting topic.

## 6. Accuracy of Multiple Tests

This section introduces methods to assess the accuracy of the combination of two or more tests. Two tests for the diagnosis of a disease measure different aspects or characteristics of the same disease. In the case of diagnostic imaging, two modalities have different qualities (resolution, contrast, and noise), thus although they are imaging the same scene, the information is not the same from the two sources. When this is the case, the accuracy of the combination of two modalities is of paramount importance. For example, the accuracy of the combination of mammography and scintimammography, for suspected breast cancer, has been reported by Buscombe, Cwikla, Holloway, and Hilson [15]. Another study for diagnosing breast cancer was performed by Berg, Gutierrez, *et al.* [16] who measured the accuracy of mammography, clinical examination, ultrasound, and MRI in a preoperative assessment of the disease, The accuracy of each modality and various combinations of the modalities were measured. When investigating metastasis to the lymph nodes in lung cancer, Van Iverhagen, Brakel, and Heijenbrok *et al.* [17] measured the accuracy of ultrasound and CT and the combination of two. Ultrasound conveys different information about metastasis compared to CT, but the combination of the two might provide a more accurate diagnosis than each separately. For an example of the diagnosis of head and neck cancer, Pauleit, Zimmerman, Stoffels *et al.* [18] used two nuclear medicine modalities, ^18^F-FET PET and ^18^F-FDG PET to assess the extent of the disease and estimated the accuracy of each and combined. On the other hand, Schaffler, Wolf, Schoelinast *et al.* [19] evaluated pleural abnormalities with CT and ^18^F-FDG PET and the combination of the two.

Switching from cancer to heart disease, Gerger, Coche, Pasquet *et al.* [20] used Four-Section Multi-Detector CT and 3D Navigator MR for detecting stenosis of the coronary arteries, where the accuracy of each and the combination of the two was estimated. The above examples involve binary test scores where accuracy is measured by TPF, FPF, PPV, and NPV, but when the test scores are ordinal and involve more than two possible values, or when the test scores are continuous, the accuracy is measured by the area under the ROC curve.

What is the optimal way to measure the accuracy for the combination of two binary tests? Pepe ([2], p. 268) presents two approaches: (1) believe the positive rule, or BP, where a positive test score on a subject means one or the other of the two tests is scored positive, and (2) believe the negative rule, or BN, where a subject is scored positive if both tests are scored positive. Pepe ([2], p. 268) also provides some properties about these rules, namely:

### Statement 1

- The BP rule increases sensitivity relative to the two binary tests, but increase the FPF, but by no more than the sum of the two false positive fractions, namely, *FPF*_1_ + *FPF*_2_.
- The BN rule decreases the false positive rate relative to the false positive rates of the two tests, but at the same time, decreases the sensitivity, however, the sensitivity remains above *TPF*_1_ +*TPF*_2_ −1.

For the first part on two binary tests, several examples are provided, then the idea is generalized to two binary tests with several readers and to two binary tests when verification bias is present. For the section on two ordinal tests, the accuracy of the combination of the two tests is provided by the ROC curve, which in turn depends on the risk score of the component tests.

This section will employ a Bayesian approach to estimate the accuracy to two binary tests and the accuracy of the combination of the two using the believe the positive BP rule and believe the negative or BN. Label the two tests *Y*_1_ and *Y*_2_ where both take on the values 0 or 1, where 0 indicates a snegative test and 1 a positive score for the medical test. A subject either has the disease or does not, as determined by the gold standard, thus when D = 1, let

(45) $$\theta_{ij}=P[Y_{1}=i,Y_{2}=j]$$

for i, j = 0,1, and when D = 0, let

(46) $$\phi_{ij}=P[Y_{1}=i,Y_{2}=j]$$

Thus the thetas are the four cell probabilities for the diseased subjects and the corresponding phis are the cell probabilities for the non-diseased subjects. The corresponding cell frequencies are denoted by *n_ij_* and *m_ij_* for the diseased and non-diseased subjects respectively, thus assuming a uniform prior for the cell probabilities, the posterior distribution of the cell probabilities are Dirichlet for *θ* = (*θ*_00_, *θ*_01_, *θ*_10_, *θ*_11_) with parameter (*n*_00_ + 1, *n*_01_ + 1, *n*_10_ + 1, *n*_11_ +1), and for *ϕ* = (*ϕ*_00_, *ϕ*_01_, *ϕ*_10_, *ϕ*_11_) is also Dirichlet with parameter (*m*_00_ +1, *m*_01_ + 1, *m*_10_ +1, *m*_11_ +1).

Once the posterior distribution of the cell probabilities is determined, the posterior distribution of the truncated cell probabilities is easily found. The truncated cell probabilities for the diseased subjects are given by

(47) $$\theta_{ij}^{*}=\theta_{ij}/\sum_{i=0}^{i=1}\theta_{ij}$$

and for the non-diseased subjects the truncated cell probabilities are

(48) $$\phi_{ij}^{*}=\phi_{ij}/\sum_{i=0}^{i=1}\phi_{ij}$$

for i and j = 0 or 1.

The true and false positive fractions for the first test *Y*_1_ are

(49) $$\text{tpf}1=\theta_{1.}$$

and

(50) $$\text{fpf}1=\phi_{1.}$$

respectively.

while for the second test the true and false positive fractions are

(51) $$\text{tpf}2=\theta_{.1}$$

and

(52) $$\text{fpf}2=\phi_{.1}$$

respectively.

The above give the accuracy of the individual tests, but what about the combination of the two? Recall there are two ways to measure the accuracy of combined tests, either by the BP rule, or by the BN rule. With the former rule, the true positive fraction is

(53) $$\text{tpfbp}=\theta_{01}+\theta_{11}+\theta_{10}$$

and the false positive fraction is

(54) $$\text{fpfbp}=\phi_{01}+\phi_{11}+\phi_{10}.$$

On the other hand, using the BN rule the true positive fraction is

(55) $$\text{tpfbn}=\theta_{11}$$

while the false positive fraction is

(56) $$\text{fpfbn}=\phi_{11}$$

In what is to follow the accuracies of the individual tests and the combined test will be estimated for several examples. The next example is based on the study of Gerber, Coche, Pasquet *et al.* [20] which investigated the use of both CT and MRI to determine the degree of stenosis in the coronary arteries, where 26 patients were suspected of having coronary artery disease. The gold standard is coronary catherization, which found 58 diseased segments (stenosis greater than 50%) and 236 non-diseased segments. This was an experimental study to determine the value of the two non invasive imaging modalities to diagnose coronary artery disease. The study found that the sensitivity of CT and MRI were 79% and 62% respectively, and that on the other hand the specificity of CT and MRI were 71% and 84% respectively, thus, CT had higher sensitivity but smaller specificity compared to MRI. This is a very interesting study and only a brief synopsis is given here, thus the reader is invited to read the article for more detail in order to know the value of the investigation. The information for the study is given below:

Our goal is to determine the accuracy of the combined test using the BP and BN rules, where the simulation consists of generating 25,000 observations from the joint posterior distribution24.

Which rule, the BP or BN rule, should be used to measure the accuracy of the combined test? Note the true positive fraction with the BP rule is higher than that with the NP rule, but on the other hand, the false positive rate is lower with the BN rule compared to the BP rule. This is a true quandary and it is not obvious which rule should be used to measure the accuracy of the combined test. Note the posterior mean for the bnfpf (believe the negative false positive fraction) is 0.1627 with a standard deviation of 0.0236. What is the best way to measure the combined tests of CT and MRI?

A change of emphasis from binary to ordinal and continuous test scores brings us to some ‘new’ ideas for measuring the accuracy by combining two tests. For ordinal and continuous scores the area under the ROC curve measures the intrinsic accuracy of a medical test, but how should the area be computed when two tests are combined? The ROC curve of the risk score is the foundation for measuring the accuracy for the combined test, but in turn, the risk score is a monotone increasing function of the likelihood ratio, which is the optimal way to measure accuracy for the combined test.

The optimality of the risk function is a consequence of the Neyman-Pearson lemma, which is a familiar result from classical statistics for testing hypotheses. In what is to follow, the likelihood ratio will be defined and the optimality of the ROC curve of the likelihood ratio will be demonstrated by referring to the Neyman-Pearson lemma, then the risk function will be defined and shown to a monotone increasing function of the likelihood ratio, thus the ROC curve of the risk function is the same as the ROC curve of the likelihood ratio. The Pepe *et al.* ([2], pp. 269–274) development of the subject is closely followed but given a Bayesian emphasis, and the end result will be that the optimal way to measure the accuracy of the combined test is to estimate the area under the ROC curve of the risk function. Determining the risk function is equivalent to performing a logistic regression using the test scores of the two tests as predictors, then the ROC curve of the predicted probabilities(from the logistic regression) is computed, from which the area is then estimated. Such an area is the accuracy of the combined test, and the methodology is illustrated with various examples using ordinal test scores. The first example is from an imaging trial using MRI and CT to detect lung cancer, where the one radiologist uses a five point confidence score, and the ROC curve of the risk function of the combined test is computed and compared to the ROC curve of the individual tests.

This section is continued with the definition of the likelihood ratio and concluded with the definition of the risk score.

Suppose *Y* = (*Y*_1_, *Y*_2_, …, *Y_p_*) is the vector of scores of p ordinal tests, then the likelihood ratio is

(57) LR(Y)=P[Y∣D=1]/P[Y∣D=0]

where D is the indicator of disease. The numerator is the probability of the observed test scores, give the disease is present, and the denominator is the probability of the observed scores, given the disease is not present.

Recall that the likelihood ratio is used as a test statistic for the null hypothesis

H: D =1

*versus* the alternative hypothesis

A: D = 0,

where larger values of LR(Y) are evidence of the null hypothesis, and smaller values are evidence the alternative is true.

It can be shown the likelihood ratio has certain optimal properties, summarized by the result:

### Statement 2

Suppose a decision about the accuracy of a medical test is based on the criterion

(58) LR(Y)>c

Then the likelihood ratio

- maximizes the TPF among all rules with FPF = t, for all *t* ∈ (0,1),
- minimizes the FPF among all rules with the TPF = r, for all *r* ∈ (0,1),
- minimizes the overall misclassification probability *ρ*(1 − *TPF*) + (1 − *ρ*)*FPF*, where *ρ* is the disease rate, and
- minimizes the expected cost, regardless of the costs associated with false negative and false positive errors.

The threshold c above appearing in Statement 2, depends on the objective at hand, but for our purposes, the above result implies the ROC curve based on the likelihood ratio is optimal, in the sense its area is the largest. The likelihood function is difficult to work with because of the complexity of determining its distribution, but, fortunately, the risk score

(59) RS(Y)=P[D=1∣Y]

does not have this disadvantage and has the property that it is a monotone function of the likelihood ratio. Simply stated, the risk score assigns a probability of disease to each study subject.

### Statement 3

The risk score has the same ROC curve as the likelihood ratio and has the same optimal properties as the likelihood ratio.

Observe that

(60) $$\begin{array}{l} \text{RS}(\text{Y})=\text{P}[\text{D}=1|\text{Y}]\\ =\text{P}[\text{Y}|\text{D}=1]\text{P}[\text{D}=1]/\text{P}[\text{Y}]\\ =\text{P}[\text{Y}|\text{D}=1]\text{P}[\text{D}=1]/\{\text{P}[\text{Y}|\text{D}=1]\text{P}[\text{D}=1]+\text{P}[\text{Y}|\text{D}=0]\text{P}[\text{D}=0]\}\\ =\text{LR}(\text{Y})\text{P}[\text{D}=1]/\{\text{LR}(\text{Y})\text{P}[\text{D}=1]+\text{P}[\text{D}=1]\} \end{array}$$

which shows that the risk score is a monotone increasing function of the likelihood ratio, which implies that the ROC curve of risk score is the same as that of the likelihood ratio. For our purposes the risk score will be used to measure the accuracy of combined tests, namely, using the area of the ROC curve of the risk score. Pepe ([2], pp. 274–275) shows the utility of logistic regression for finding the ROC curve of the risk score. Note, that the following statement show why.

### Statement 4

Suppose the risk score is expressed as

(61) logitP[D=1∣Y]=γ+g(λ,Y)

where g is a known function, then:

(a) the parameter *λ* can be estimated, even for retrospective designs in which the sampling depends son D, and (b) the function g is optimal for determining the ROC curve of the risk function.

From a practical point of view, logistic regression can be used to determine the ROC curve of the risk function, but it should be noted that finding a suitable function g can be challenge. After all, g can be a complicated non linear function of *λ* and/ or Y, but it would be convenient if g is linear in the test scores Y. Of course, a Bayesian approach is taken in order to estimate the logistic regression function (10.48).

The approach taken here is based on the risk score and Pepe ([2], pp. 274–275) gives a good account.

Suppose there are two medical tests with ordinal scores, then for diseased subjects the layout is:

Thus, there are *n_ij_* diseased subjects with a score of i for test 1 and score j for test 2 and the cell probabilities for the diseased are

(62) $$\theta_{ij}=P[T_{1}=i,T_{2}=j|\text{D}=1]$$

for the first test, where i, j = 1,2,…,k.

The non-diseased cell probabilities are

(63) $$\phi_{ij}=P[T_{1}=i,T_{2}=j|\text{D}=0]$$

Define the ROC area for test 1, the usual way, as:

(64) $$\text{Area}1=A_{11}+A_{12}/2$$

where

(65) $$A_{11}=\sum_{i=1}^{i=k}\theta_{i.}(\sum_{j=1}^{j=i-1}\phi_{j.})$$

and the *θ_i_*., i = 1,2,…,k, are the sum of the *θ_ij_* over the missing subscript.

and

(66) $$A_{12}=\sum_{i=1}^{i=k}\theta_{i.}\phi_{i.}$$

The ROC area for the second test is defined in a similar fashion as

(67) $$\begin{array}{c} \text{Area}2=A_{21}+A_{22}/2\\ A_{12}=\sum_{i=2}^{i=k}\theta_{.i}(\sum_{j=1}^{j=i-1}\phi_{.j})\\ \text{and}\\ A_{22}=\sum_{i=1}^{i=k}\theta_{.i}\phi_{.i} \end{array}$$

Our goal is to use the area under the ROC of the risk score as a measure of accuracy of the combined tests *T*_1_ and *T*_2_, where the risk scores are determined by logistic regression (if appropriate)

(68) $$\log\mathrm{it}(\theta_{ij})=\gamma +g(\lambda ,T_{1},T_{2})$$

and the unknown parameters *γ* and *λ*, (possibly a vector) are estimated by Bayesian techniques. From the logistic regression, the estimated (e.g. posterior means) cell probabilities are employed to estimate the area under the ROC curve of the risk score.

Note that the area under the ROC curve of the risk score is based on the posterior distribution of the 2*k*^2^ parameters *θ_ij_* and *ϕ_ij_* for i, j = 1,2,…,k, and this scenario is illustrated with the following example, where the area under the ROC curve is given by the usual formulas employed in earlier in sections. Of course, in addition the area under the ROC curves for the individual tests will also be portrayed and compared to the area under the ROC curve of the risk score. It will be a challenge to develop a good logistic regression, however, in some cases it will turn out that the logit is a linear function of the two tests *T*_1_ and *T*_2_. The risk score is assigned to each experimental unit and is the probability of disease, which is estimated from the raw scores of the two component tests! Note, using the risk score is a statistical procedure and will ideally be utilized by the clinician working with a statistician.

When considering the accuracy of two ordinal tests, a paired study is envisioned, where each test is applied to each patient and one reader examines the results of both tests. It is important to remember that the reader uses the results of both tests for each patient in order to decide what score to assign to the patient.

Our first example involves the MRI and CT determination of the lung cancer risk, where one radiologist interprets both images and gives a score from 1–5 for the presence of a malignant lesion with the following definition: A score of 1 indicates no evidence of malignancy, while a score of 2 indicated very little evidence of a lesion. The score of 3 designates a benign lesion, while a score of 4 indicates there is some evidence of a malignancy, and finally a score of 5 signals that the lesion is definitely malignant. This is obviously a paired design in that both images are taken on each patient and one would expect a ‘large’ correlation between the scores of MRI and CT images. There are 261 patients that have lung cancer and 674 who do not, and the gold standard is lung biopsy.

The above study is hypothetical, but there are many studies that have investigated CT and MRI as alternatives to detecting lung cancer, and it should be noted that CT has shown good promise (in comparison to X-ray) in a recent national lung cancer screening trial, see Gierada, Pilgrim, Ford *et al.* [21] for additional information.

With regard to the accuracy of the combined test, the approach is to find the area under the ROC curve of the risk score, which is determined by logistic regression, namely,

(69) $$\log\mathrm{it}(\text{theta}[i])=b[1]+b[2]T_{1}[i]+b[3]T_{2}[i]$$

where theta[i] is the probability the i-th patient has disease, where i = 1,2,…,N.

N is the number of patients in the study with 261 with disease (lung cancer) and 674 with no disease, and the *b*[*i*] are unknown regression coefficients. From a Bayesian viewpoint, the regression coefficients are given vague prior distributions of the form

(70) b[i]~dnorm(.000,.0001)

namely, a normal distribution with mean 0 and precision 0.0001.

Based on generating 45,000 observations generated from the posterior distribution, the Bayesian analysis is presented below.

The MCMC errors are quite small and show that the presented estimated ROC areas are very ‘close’ to the actual posterior areas, and the analysis also shows that the two areas are about the same, that is the accuracy of the two modalities are essentially the same. The probability of a tie with CT is estimated with a posterior mean of 0.181 and 0.1788 with MRI. Thus one would expect the accuracy of the combined test, as measured by the ROC area of the risk score, to be about the same value, in the area of 0.70.

As before, when estimating the ROC area of the risk score, 45,000 observations are generated for the MCMC simulation, with the following results.

The auc parameter is the ROC area of the risk score and is estimated as 0.7246(0.0192) with the posterior mean, and the median is about the same value indicating very little skewness in the posterior distribution. The implication is that the combined test has an accuracy is somewhat larger the accuracy of the individual tests, see Table 27 which portrays the individual area as approximately 0.68. Of course, this is not surprising because the individual ROC area for CT and MRI are essentially the same, thus, one would expect the accuracy of the combined test to be about the same as the individual values.

Note, that the b's are the regression coefficients for the logistic regression, and the beta's are the regression coefficients in the normal regression for the ROC area of the risk score. The logistic regression is linear in the two test variables *T*_1_ and *T*_2_, but I did add the squares and cross product of the two and the ROC area remained the same, thus, the linear association appears to be adequate for estimating the risk score for the combined test. The risk scores are not normally distributed, but can be transformed to normality approximately via the log transformation, however, when this is done the ROC area remains at about 0.72.

Of course there are examples, where the ROC area of the risk score is much greater than that of the component tests. A good example is one for a pancreatic cancer study analyzed by Pepe [2, p9]] who investigated the effect of two biomarkers on the disease incidence. The first biomarker is CA19-9 and the second biomarker is CA125. On the original scale the mean(sd) of CA19-9 is 18.03(20.81) for the 51 control patients and 1715(3681) for the cancer patients, whereas for the CA125 marker, the mean(sd) for the control patients are 21.81(30.29) and 55.04(138.8) for the diseased.

The median for the first biomarker is 10 for the control and 249 for the cancer patients, and for the second biomarker, the medians are 11.4 *versus* 21.8 for the control and diseased patients respectively. Note the large variability of both biomarkers, but based on the difference in the means and medians between the diseased and non-diseased patients, one would expect a high value for the ROC area of CA19-9. Note that *T*_1_ is the CA19-9 biomarker, *T*_2_ is CA125. In order to determine the accuracy of the combined test, the Bayesian analysis is executed with 45,000 observations, and the results reported in Table 30.

Using the risk score (which is determined with a logistic regression that regresses the disease status on the logs of the two biomarkers *T*_1_ and *T*_2_) a ROC area with posterior mean 0.912 implies very sgood accuracy for the combined test. This is to be compared to an ROC area of 0.8733(0.0275), based on CA19-9, and 0.6786(0.0438) for CA125.

Bayesian Methods for determining the accuracy of combined tests can easily be extended to other situations and the reader is referred to Broemeling [1].

## 7. Comments and Conclusions

The article has described some of the Bayesian methods that are available for determining the accuracy of medical tests and began with the basic measure of accuracy including the true and false positive fractions and the positive and negative predictive values. For ordinal and continuous test scores, Bayesian methods for estimating the ROC area were introduced. The review was continued by considering more specialized scenarios, including studies where verification bias is present and where an imperfect reference standard is used, and for each scenario the methodology was illustrated with interesting examples that occur in cancer and other diseases.

Other scenarios were not considered but nevertheless are important topics for Bayesian methods of medical test accuracy. One important topic not covered is the subject of multiple observers, each providing an estimate of test accuracy. Consider the case where two radiologists are interpreting the same mammograms for diagnosing breast cancer, then how does one resolve any differences in interpretation and to what degree do the observers agree in their interpretation? This topic is studied from a Bayesian viewpoint in some detail by Broemeling [23], where the various analyses are executed with the WinBUGS package. Another area not presented in this review is that of Bayesian nonparametric inference, thus, the reader is referred to Erkanli *et al.* [24] and Hanson *et al.* [25] for additional information on this approach to medical test accuracy. Also absent from this review are certain aspects of the design of accuracy studies, therefore, for a good introduction refer to Dendukuri *et al.* [26] and Cheng *et al.* [27].

## Figures and Tables

**Figure 1:**
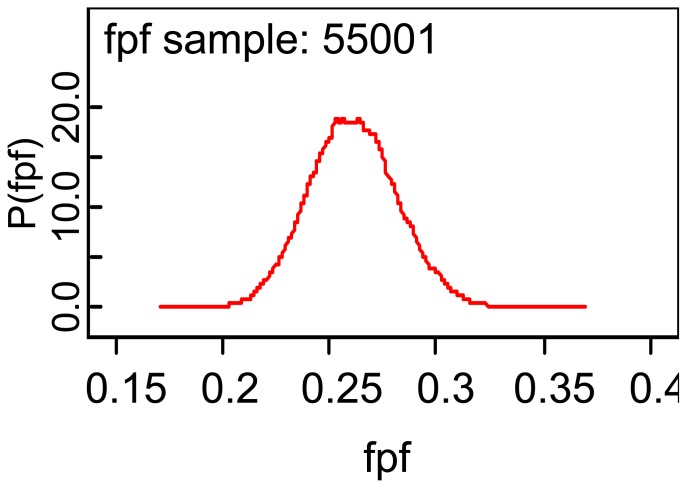
Posterior density of the false positive fraction.

**Table 1:** Classification table.

**Test**	**D = 0**	**D = 1**
X = 0	(*n*_00_, *θ*_00_)	(*n*_01_, *θ*_01_)
X = 1	(*n*_10_,*θ*_10_)	(*n*_11_, s*θ*_11_)

**Table 2:** Exercise stress test and heart disease.

**EST**	**D = 0**	**D = 1**
X = 0	327	208
X = 1	115	818

**Table 3:** Posterior distribution of the true and false positive fractions.

**Parameter**	**Mean**	**SD**	**Error**	**Lower 2 1/2**	**Median**	**Upper 2 1/2**
TPF	0.7967	0.0125	5.84 × 10^−5^	0.7716	0.7968	0.8208
FPF	0.2612	0.0208	9.22 × 10^−5^	0.2215	0.2608	0.3033

**Table 4:** Posterior distribution of predictive values.

**Parameter**	**Mean**	**SD**	**Error**	**Lower 2 1/2**	**Median**	**Upper 2 1/2**
PPV	0.8759	0.0108	<0.0001	0.8538	0.8762	0.8961
NPV	0.6109	0.0211	<0.0001	0.5693	0.611	0.6517

**Table 5:** Posterior distribution of diagnostic likelihood ratios.

**Parameter**	**Mean**	**SD**	**Error**	**Lower 2 1/2**	**Median**	**Upper 2 1/2**
PDLR	3.07	0.2526	0.0011	2.616	3.055	3.609
NDLR	0.2755	0.0187	<0.0001	0.2399	0.275	0.3135

**Table 6:** Mammogram results.

**Status**	**Normal 1**	**Benign 2**	**Probably Benign 3**	**Suspicious 4**	**Malignant 5**	**Total**
Cancer	1	0	6	11	12	30
No Cancer	9	2	11	8	0	30

**Table 7:** TPF *versus* FPF for Mammography.

**Status**	**Normal 1**	**Benign 2**	**Probably Benign 3**	**Suspicious 4**	**Malignant 5**
tpf	30/30 = 1.00	30/30 = 1.00	29/30 = 0.966	23/30 = 0.766	12/30 = 0.400
fpf	30/30 = 1.00	21/30 = 0.700	19/30 = 0.633	8/30 = 0.266	0/30 = 0.000

**Table 8:** Posterior distribution of area under the ROC curve.

**Parameter**	**Mean**	**SD**	**Error**	**Lower 2 1/2**	**Median**	**Upper 2 1/2**
auc	0.7811	0.0514	<0.001	0.6702	0.7848	0.8709
A1	0.688	0.0635	<0.001	0.5564	0.6909	0.8036
A2	0.1861	0.0307	<0.001	0.128	0.1854	0.2484

**Table 9:** Posterior distribution for the ROC area.

**Parameter**	**Mean**	**SD**	**Error**	**Lower 2 1/2**	**Median**	**Upper 2 1/2**
beta [1]	107.5	2.237	0.0401	103	107.5	111.9
beta [2]	16.14	2.438	0.2438	11.35	16.11	21.02
precy [1]	0.0129	0.004	<0.0001	0.00544	0.0116	0.0211
precy [2]	0.0205	0.0038	<0.0001	0.0137	0.0203	0.0286
auc	0.9084	0.04227	<0.0001	0.8062	0.9155	0.9689

**Table 10:** **(a)** CT and MRI study for diseased subjects; **(b)** CT and MRI study for non-diseased subjects.

**(a)**			
**CT**	**MRI=0**	**MRI=1**	**TOTAL**
0	22	191	213
1	30	752	782
TOTAL	52	943	995

**(b)**			
**CT**	**MRI=0**	**MRI=1**	**TOTAL**
0	148	167	315
1	49	71	120
TOTAL	197	238	435

**Table 11:** Posterior analysis for CT and MRI imaging for lung cancer.

**Parameter**	**Mean**	**SD**	**Error**	**2 1/2**	**Median**	**97 1/2**
fpfct	0.2778	0.0212	<0.00001	0.2375	0.2775	0.3202
fpfmri	0.5468	0.2371	<0.0001	0.5	0.5469	0.5928
rfpf(ct/mri)	0.5089	0.0437	<0.0001	0.427	0.5076	0.5984
rtpf(ct/mri)	0.8295	0.0144	<0.00001	0.801	0.8296	0.8577
tpfct	0.7847	0.0130	<0.00001	0.7589	0.7848	0.8097
tpfmri	0.9595	0.0071	<0.00001	0.931	0.9462	0.9592

**Table 12:** One binary test.

**V=1**		**Y=1**	**Y=0**
	D=1	*s*_1_	*s*_0_
	D=0	*r*_1_	*r*_0_
V=0		*u*_1_	*u*_0_
Total		*m*_1_	*m*_0_

**Table 13:** Hepatic scintigraphy study for verification bias.

V=1		Y=1	Y=0
	D=1	*s*_1_ = 298	*s*_0_ = 31
	D=0	*r*_1_ = 26	*r*_0_ = 48
V=0		*u*_1_ = 150	*u*_0_ =117
Total		*m*_1_= 474	*m*_0_= 196

**Table 14:** Posterior distribution for hepatic scintigraphy study.

**Parameter**	**Mean**	**SD**	**Error**	**2 1/2**	**Median**	**97 1/2**
fpf	0.2139	0.0336	<0.0001	0.1525	0.2125	0.2834
tpf	0.8393	0.0235	<0.0001	0.7918	0.84	0.8836

**Table 15:** Verification bias and one ordinal test.

**V = 1**	**Y = 1**	**Y = 2**	**Y = k**
D = 1	*s*_1_	*s*_2_	*s_k_*
D = 0	*r*_1_	*r*_2_	*r_k_*
V = 0	*u*_1_	*u*_2_	*u_k_*
Total	*m*_1_	*m*_2_	*m_k_*

**Table 16:** Ordinal results for mammography.

**V = 1**	**Y = 1**	**Y = 2**	**Y = 3**	**Y = 4**	**Y = 5**
D = 1	*s*_1_ = 72	*s*_2_ = 54	*s*_3_ = 121	*s*_4_ = 145	*s*_5_ = 245
D = 0	*r*_1_ = 308	*r*_2_ = 127	*r*_3_ = 78	*r*_4_ = 33	*r*_5_ = 77
V = 0	*u*_1_ = 92	*u*_2_ = 66	*u*_3_ = 76	*u*_4_ = 10	*u*_5_ = 5
Total	*m*_1_ = 472	*m*_2_ = 247	*m*_3_ = 275	*m*_4_ = 188	*m*_5_ = 327

**Table 17:** Posterior analysis for mammography study.

**Parameter**	**Mean**	**SD**	**Lower 2 1/2**	**Median**	**Upper 2 1/2**
*A*	0.7762	0.0126	0.7509	0.7764	0.8005
*A*_1_	0.6972	0.0154	0.6665	0.6974	0.7272
*A*_2_	0.0789	0.00303	0.0729	0.0789	0.0848

**Table 18:** Hypothetical example imperfect reference.

**New Test**	**D = 0**	**D = 1**	**R = 0**	**R = 1**
T = 0	70	20	74	16
T = 1	30	80	46	64
Total	100	100	120	80

**Table 19:** **(a)** Augmented data for reference R and test T when D = 1; **(b)** Augmented data for R and T, when D = 0.

**(a)**		
**Reference Test**	**T = 1**	**T = 0**
R = 1	y_11_	y_10_
R = 0	y_10_	y_00_
Total		

**(b)**		
**Reference Test**	**T = 1**	**T = 0**
R = 1	*n*_11_ − y_11_	*n*_10_ − y_10_
R = 0	*n*_01_ − y_10_	*n*_00_ − y_00_
Total		

**Table 20:** Results of a stool exam T and a serologic reference exam R.

**Serology Test R**	**T = 1**	**T = 0**	**Total**
R = 1	38	87	125
R = 0	2	35	37
	40	122	162

**Table 21:** Posterior analysis for the stool and serology exams.

**Parameter**	**Mean**	**SD**	**Error**	**2 1/2**	**Median**	**97 1/2**
p	0.4986	0.2011	0.0053	0.1604	0.4991	0.8352
*c*_1_	0.6977	0.2586	0.0109	0.0922	0.7046	0.9942
*c*_2_	0.2504	0.2552	0.0109	0.0042	0.1237	0.8862
*s*_1_	0.2517	0.2503	0.0107	0.0046	0.1308	0.8826
*s*_2_	0.7014	0.2623	0.0109	0.0889	0.7192	0.9945

**Table 22:** Prior information about stool and serology tests.

**Parameter**	**Range**	**Alpha**	**Beta**
p	0–100	1	1
*c*_1_	90–100	71.25	3.75
*c*_2_	35–100	4.1	1.76
*s*_1_	5–45	4.44	13.31
*s*_2_	65–95	21.96	5.49

**Table 23:** Posterior analysis for the stool and serology exams.

**Parameter**	**Mean**	**SD**	**Error**	**2 1/2**	**Median**	**97 1/2**
p	0.7618	0.1007	0.0014	0.5236	0.7755	0.9286
*c*_1_	0.957	0.0214	<0.0001	0.9065	0.9603	0.9885
*c*_2_	0.6901	0.1605	0.0020	0.3727	0.7006	0.9558
*s*_1_	0.3093	0.0518	<0.0001	0.2224	0.3043	0.4269
*s*_2_	0.8831	0.0423	<0.0001	0.7892	0.8874	0.9535

**Table 24:** **(a)** Study results the CT-MRI study; **(b)** study results the CT-MRI study.

**(a)**			
**CT**	**MRI=0**	**MRI=1**	**Total**
0	12	0	12
1	10	36	46
Total	22	36	58

**(b)**			
**CT**	**MRI=0**	**MRI=1**	**Total**
0	168	0	168
1	30	38	68
Total	198	38	236

**Table 25:** Bayesian analysis for combined test of CT and MRI.

**Parameter**	**Mean**	**SD**	**Error**	**2 1/2**	**Median**	**97 1/2**
bnfpf	0.1627	0.0236	<0.0001	0.1191	0.1617	0.2116
bntpf	0.5965	0.0623	<0.0001	0.4731	0.5977	0.7148
bpfpf	0.2959	0.0293	<0.0001	0.2404	0.2953	0.3553
bptpf	0.7901	0.0515	<0.0001	0.68	0.7933	0.8817
fpfct	0.2918	0.0292	<0.0001	0.2361	0.2911	0.3511
fpfmri	0.1668	0.0238	<0.0001	0.1228	0.1658	0.2163
tpfct	0.7739	0.0527	<0.0001	0.662	0.766	0.8675
tpfmri	0.6127	0.0619	<0.0001	0.4883	0.614	0.7299

**Table 26:** **(a)** Two medical tests for diseased patients: frequencies and probabilities; **(b)** two medical tests for non-diseased patients: frequencies and probabilities.

**(a)**							
**Test 1**	**Test 2=1**	**Test 2=2**		.	.	.	**Test 2=k**
1	*n*_11_, *θ*_11_	*n*_12_, *θ*_12_					*n*_1_*_k_, θ*_1_*_k_*
2	*n*_21_, *θ*_21_	*n*_22_, *θ*_22_					*n*_2_*_k_, θ*_2_*_k_*
	.						
	.						
k	*n_k_*_1_, *θ_k_*_1_	*n_k_*_2_, *θ_k_*_2_					*n_kk_, θ_kk_*

**(b)**							
**Test 1**	**Test 2=1**		**Test 2 =2**	.	.	.	**Test 2=k**
1	*m*_11_, *ϕ*_11_		*m*_12_,*ϕ*_12_				*m*_1_*_k_, ϕ*_1_*_k_*
2	*m*_21_, *ϕ*_21_		*m*_22_, *ϕ*_22_				*m*_2_*_k_, ϕ*_2_*_k_*
.							
.							
k	*m_k_*_1_, *ϕ_k_*_1_		*m_k_*_2_, *ϕ_k_*_2_				*m_kk_, ϕ_kk_*

**Table 27:** **(a)** MRI and CT scores for diseased patients; **(b)** MRI and CT scores for non-diseased patients.

**(a)**						
**CT Scores**	**MRI = 1**	**MRI = 2**	**MRI = 3**	**MRI = 4**	**MRI = 5**	**Total**
1	15	10	6	2	1	34
2	9	21	10	3	2	45
3	5	6	32	6	3	52
4	2	0	6	47	2	57
5	0	1	2	5	65	73
Total	31	38	56	63	73	261

**(b)**						
**CT Scores**	**MRI = 1**	**MRI = 2**	**MRI = 3**	**MRI = 4**	**MRI = 5**	**Total**
1	92	62	41	8	5	208
2	58	81	10	8	4	161
3	38	30	65	31	18	182
4	16	2	21	35	12	86
5	5	1	3	11	17	37
Total	209	176	140	93	56	674

**Table 28:** Posterior analysis for MRI and CT of individual ROC areas.

**Parameter**	**Mean**	**SD**	**Error**	**2 1/2**	**Median**	**97 1/2**
area ct	0.6836	0.0188	<0.0001	0.6459	0.6837	0.7198
area11	0.5931	0.0213	<0.0001	0.5509	0.5931	0.6346
area12	0.181	0.0057	<0.0001	0.1696	0.1811	0.1921
area mri	0.6886	0.0183	<0.0001	0.652	0.6889	0.7239
area21	0.5992	0.0207	<0.0001	0.5581	0.5994	0.6392
area22	0.1788	0.0049	<0.0001	0.1689	0.1789	0.1883

**Table 29:** Posterior accuracy of the combined test.

**Parameter**	**Mean**	**SD**	**Error**	**2 1/2**	**Median**	**97 1/2**
auc	0.7246	0.0192	<0.0001	0.6858	0.725	0.7616
b [1]	−2.952	0.2162	<0.0001	−3.381	−2.949	−2.533
b [2]	0.3562	0.0752	<0.0001	0.2089	0.3562	0.504
b [3]	0.3392	0.0723	<0.0001	0.1965	0.3391	0.481
beta [1]	0.2412	0.0053	<0.0001	0.2309	0.2412	0.2516
beta [2]	0.1385	0.0127	<0.0001	0.1136	0.1385	0.1638
precy [1]	53.13	2.904	0.0143	47.59	53.1	58.92
precy [2]	28.79	2.528	0.0122	24.08	28.71	33.99

**Table 30:** Bayesian analysis for accuracy of the combined test.

**Parameter**	**Mean**	**SD**	**Error**	**2 1/2**	**Median**	**97 1/2**
auc	0.9127	0.0227	<0.0001	0.8625	0.915	0.951
beta [1]	0.3568	0.0223	<0.0001	0.3129	0.3567	0.4015
beta [2]	0.4361	0.0368	<0.0001	0.3633	0.4362	0.5084
